# Patient and surrogate attitudes via an interviewer-administered survey on exception from informed consent enrollment in the Prehospital Air Medical Plasma (PAMPer) trial

**DOI:** 10.1186/s12873-020-00371-6

**Published:** 2020-10-01

**Authors:** Insiyah Campwala, Francis X. Guyette, Joshua B. Brown, Peter W. Adams, Barbara J. Early, Mark H. Yazer, Matthew D. Neal, Brian S. Zuckerbraun, Jason L. Sperry

**Affiliations:** 1 Division of Trauma and General Surgery, Department of Surgery, University of Pittsburgh, 200 Lothrop St, Pittsburgh, PA 15213 USA; 2 Department of Emergency Medicine, University of Pittsburgh, Pittsburgh, PA USA

**Keywords:** Exception from informed consent, Emergency research, Hemorrhagic shock, Patient/SDM attitudes, Telephone survey

## Abstract

**Objectives:**

With increased focus on early resuscitation methods following injury to improve patient outcomes, studies are employing exception from informed consent (EFIC) enrollment. Few studies have assessed patients’ opinions following participation in an EFIC study, and none have been conducted within the realm of traumatic hemorrhage. We surveyed those patients and surrogates previously enrolled in the Prehospital Air Medical Plasma (PAMPer) Trial to clarify their opinions related to consent and emergency research.

**Methods:**

Telephone calls were made between January–June 2019 to all patients who were enrolled under EFIC in the PAMPer study at the Pittsburgh site (169 of the 501 total patients enrolled, May 2014-Oct 2017) and their surrogates. Questions gauging approval of EFIC enrollment were asked before discussion of PAMPer trial outcomes, after disclosure of positive outcomes, and after a hypothetical negative trial outcome was proposed.

**Results:**

Of the total 647 telephone calls made, ninety-three interviews, reflecting 70 of 169 patient enrollments, were conducted. This included 13 in which only the patient was interviewed, 23 in which the patient and a surrogate were interviewed, and 34 in which only a surrogate was interviewed. Nearly half (48.4%) of respondents did not recall their personal or family member enrollment in the study. No patients or surrogates recalled hearing about the study through community consultation or being aware of opt out procedures. Patients and surrogates were glad they were enrolled (90.3%), agreed with EFIC use for their personal enrollment (88.17%), and agreed with the general use of EFIC for the PAMPer study (81.7%). Disclosure of the true positive PAMPer study outcome resulted in a significant increase in opinions regarding personal enrollment, EFIC for personal enrollment, and EFIC for general enrollment (all *p* < 0.001). Disclosure of a hypothetical neutral or negative study outcome resulted in significant decreases in opinions regarding EFIC for personal enrollment (*p* = 0.003) and EFIC for general enrollment (*p* < 0.001).

**Conclusions:**

Clinical trial participants with traumatic hemorrhagic shock enrolled with EFIC, and surrogates of such participants, are generally accepting of EFIC. The results of the trial in which EFIC was utilized significantly affected patient and surrogate agreement with personal and general EFIC enrollment.

## Background

Recent studies have found that deaths following traumatic injury occur quickly—often within the first few hours following arrival to definitive care. As a result, management of severe injury has changed over the last decade, and improvements have occurred primarily in the in-hospital setting. Packed red blood cells [1] and plasma [2] have been shown to improve survival in patients at risk of hemorrhagic shock.

In clinical trials that focus on emergency interventions, most patients are unresponsive or incapable of making informed consent decisions related to their treatment, and legally authorized representatives are often not immediately available to make decisions on their behalf. This has led to increasing use of exception from informed consent (EFIC) for such trials [3, 4]. EFIC is a method of enrollment without consent in emergencies in which consent cannot be obtained. Surrogates are notified as soon as feasible and patients and/or surrogates are consented for continued participation after patients are medically stabilized and are capable of informed decision-making.

EFIC requires that participants suffer from life-threatening disease or injury for which available treatments are unproven or unsatisfactory. The research must have the prospect of direct benefit to the patient, and the treatment is only effective if given in a window of time in which consent cannot be practicably obtained. The US Food and Drug Administration also requires a public disclosure and community consultation phase prior to beginning the trial to inform communities of the proposed study and provide ways in which individuals wishing to opt out may be excluded. It is known that the general population is largely accepting of EFIC enrollment [5–20], and community consultation practices have been associated with increased acceptance [9, 21–24]. However, there is a paucity of studies that have characterized the experiences of patients or surrogates enrolled in the actual EFIC studies [25–30], and none have been conducted following interventions that focus on traumatic hemorrhage. Whether the outcome of the trial affects patient or surrogate opinions remains poorly characterized.

We aimed to clarify patient and surrogate opinions and values related to consent and emergency research and appropriately characterize these knowledge gaps in the literature. We sought to assess patient and surrogate factors that predicted acceptance of EFIC enrollment procedures and evaluate the success of community consultation and public disclosure in bringing awareness of the PAMPer trial to the individuals enrolled. We surveyed those patients and surrogates who were enrolled in the Prehospital Air Medical Plasma (PAMPer) Trial, conducted under the guidelines of EFIC for emergency research [2]. We hypothesized that trial results and patient outcomes will influence patients’ and surrogates’ satisfaction with EFIC enrollment, and patients’ and surrogates’ views on EFIC will be similar.

## Methods

### Objective

Our principal objective was to learn about the experience of patients and surrogates who were enrolled in the PAMPer EFIC study and their general opinions on EFIC enrollment and emergency research.

### Principal study and ethical approval

The Prehospital Air Medical Plasma (PAMPer) Trial was a pragmatic, multicenter, cluster-randomized, phase 3 trial that compared the administration of thawed plasma with standard-care resuscitation during air medical transport from May 2014 to October 2017. Other than the administration of plasma, no other aspect of treatment either during patient transport or after arrival at the trauma center was altered. Prehospital administration of plasma was not part of standard care for any of the participating sites during the trial. Results showed that prehospital administration of thawed plasma resulted in a 9.8% lower mortality rate as compared to standard-care resuscitation [2].

The PAMPer trial was designed by the authors, and the Food and Drug Administration, the Human Research Protection Office of the Department of Defense, and the institutional review boards at the participating sites approved the design. The Institutional Review Board at each site approved exception from informed consent requirements, after consultation with community members and after public disclosure regarding the trial took place. Opt out bracelets were available to community members by request via email or phone. Following a trauma, enrolled participants or their legally authorized representatives were notified and asked to provide consent for continued participation as soon as feasible.

This sub-study of the PAMPer trial (STUDY18100001) was approved by the University of Pittsburgh Institutional Review Board on 12/13/2018.

### Study population

All patients who were enrolled under EFIC in the PAMPer trial at the Pittsburgh site (169 of the 501 total patients enrolled) and those surrogates who signed consent for continued participation after their family member was enrolled in this trial were eligible for this sub-study. Surrogates for patients who died after EFIC enrollment were still eligible for this survey. The first listed surrogate was contacted, but if that person could not be contacted, other surrogates were attempted. No more than one surrogate completed the survey for each enrolled subject.

### Interview methods

A survey (see Supplemental Text Document) was created to elicit the attitudes of both patients and surrogates about EFIC for emergency research and trial participation. Questions were derived from previously published questions from other surveys [5, 6, 15, 19, 25, 26]. Questions were specifically adopted from published literature in which patients and/or surrogates were interviewed after being enrolled in an EFIC study. Additionally, questions assessing overall attitudes toward emergency research and EFIC enrollment were adopted from published surveys assessing the general population’s attitudes during community consultation efforts. The survey consisted of 12 domains: 1) demographic information 2) prior research experience and general attitude toward research 3) recall of enrollment in PAMPer 4) views of acceptability of personal EFIC inclusion 4) views of acceptability of general EFIC enrollment, 5) opinions on the need for community consultation 6) prior knowledge of PAMPer via community consultation 7) prior knowledge of opt out procedures for PAMPer 8) hypothetical choice to opt out of PAMPer prior to injury and enrollment 9) opinions regarding the intentions and honesty of doctors and researchers 10) opinions on emergency research 11) views of personal enrollment, use of EFIC for personal and general enrollment, and randomization technique given a positive study outcome 12) views of personal enrollment, use of EFIC for personal and general enrollment, and randomization technique given a negative study outcome. Each domain consisted of a brief introductory statement followed by five-point Likert Scale questions (1 = strongly agree, 3 = no opinion, 5 = strongly disagree). Results were reported by collapsing responses into three categories: Agree—1 and 2, Neutral—3, and Disagree—4 and 5.

Patient and surrogate names, phone numbers, addresses, PAMPer enrollment dates, hospital length of stay, length of mechanical ventilation, injury severity score, type of trauma, and mechanism of transport to hospital were compiled from previously collected PAMPer trial data. A letter informing patients of the project was mailed out and provided them the option to opt out of receiving a phone call. A 10-min telephone survey was administered in English and began with an introductory script telling patients and surrogates about the study, its risks and benefits, and asked interviewees to verbally consent to use of questionnaire answers for the project. The introductory script also offered a second opportunity for interviewees to opt out of the study. No incentives for participation were offered. Participants’ age, race, education level, job status, income level, and prior research participation experience were compiled. Patients were asked to indicate their opinions on a series of statements related to their enrollment, general EFIC practices, and emergency research. The first part of the interview was conducted without mentioning the results of the PAMPer study and asked patients and surrogates about their opinions using a range of questions about protocols, enrollment, and emergency research overall. Following this, subjects were told the actual outcome of the PAMPer study. Then, subjects were asked to respond to select questions previously asked once again to document changes. Finally, subjects were told a hypothetical neutral or negative study outcome for the trial and asked to respond to the same select set of questions for a third time. Telephone surveys were all conducted by a single author (IC) who was blinded to which arm (plasma or standard-care resuscitation) the patient was assigned. Each patient was called at least four times at different times of the day and days of the week before being eliminated from the list, and each surrogate was called at least 2 times before moving to the next family member on the patients’ contact list. Voice messages were left for patients and surrogates after the first and third call, and a call-back number was provided.

To allow for follow-up questions or concerns to be addressed, subjects were provided with the phone number for a PAMPer study primary investigator and the University of Pittsburgh Human Subjects Protection Advocate.

### Analysis

Data storage and management was performed with Microsoft Excel (Redmond, WA) software package. Data was analyzed using SPSS Statistics, version 26, for Mac (IBM, Armonk, NY). Descriptive statistics were used to describe the patient group, the surrogate group, and total respondents. We measured the effectiveness of our public disclosure and community consultation. We then compared patient and surrogate attitudes toward EFIC and measured the effect of patient survival on surrogate views.

Chi-square was used to compare categorical variables. Binary logistic regression was used to determine the relationship between age, gender, prior research experience, respondent type, and attitudes reported via the survey. Non-parametric comparisons with independent-samples Mann-Whitney U Tests were used to compare patient and surrogate responses. A *p*-value of < 0.05 was considered to be significant for all analyses.

## Results

### Study population

**Fig. 1:**
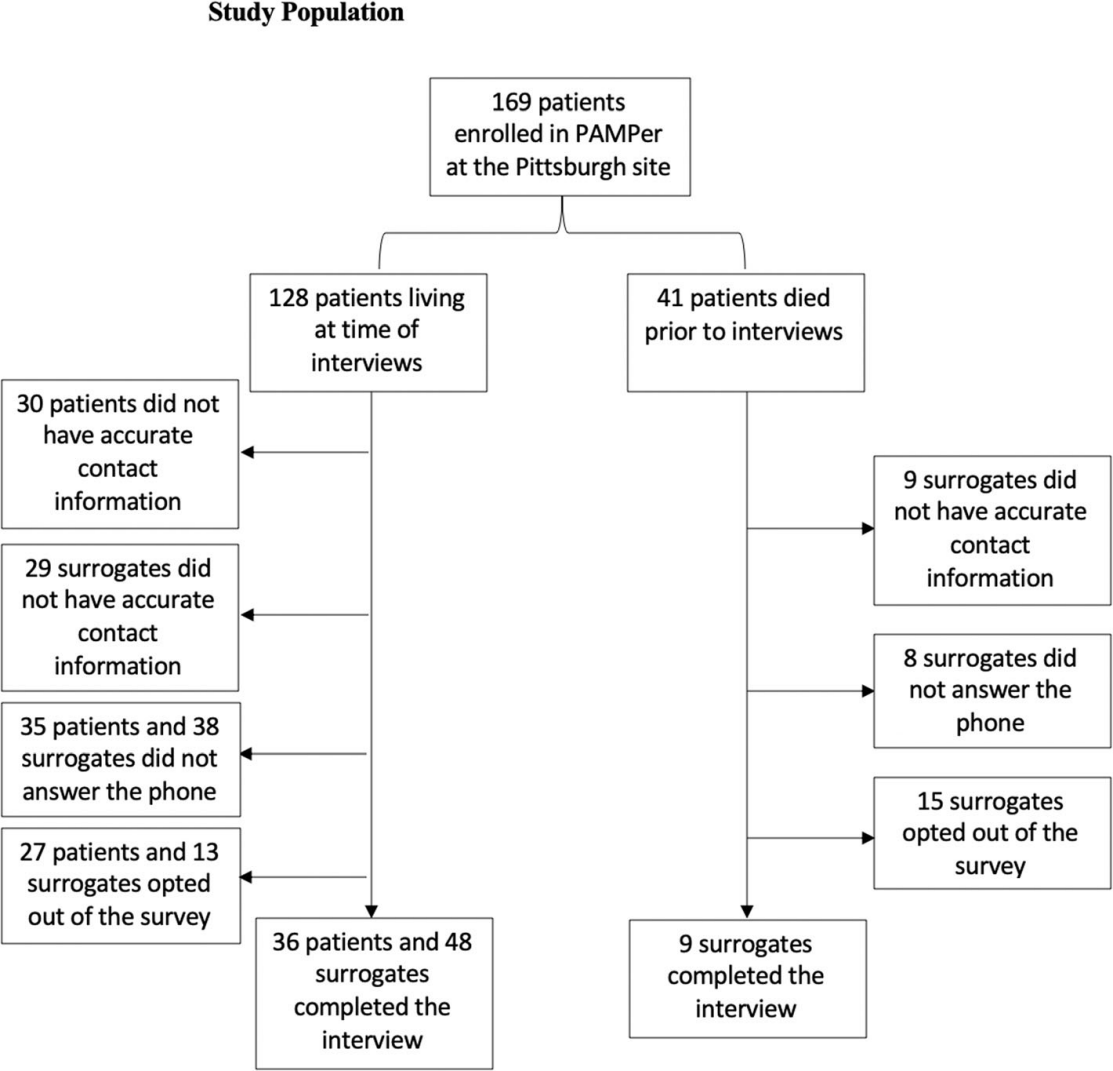
Study Population

A total of 647 telephone calls were made between January 2019 and June 2019 to all patients who were enrolled under EFIC in the PAMPer study at the University of Pittsburgh site and their surrogates. One hundred seventy-one voicemails were left for patients or surrogates. Accurate contact information was not available for 30 patients and 38 surrogates. In this Pittsburgh cohort, 41 patients died prior to this study (24.3%). After the initial letter informing patients and surrogates about that the study was mailed out, two subjects opted out of the survey. Additional participants opted out during the phone call--after the introduction was read to them; the primary reasons included refusal to participate (*n* = 32), the death of the patient being too painful to discuss (*n* = 7), lack of memory of the incident (*n* = 6), surrogates not being involved in the enrollment process (*n* = 6), and being unhappy with the hospital/their insurance (*n* = 2). Ninety-three interviews, reflecting 70 of 169 participant enrollments, were conducted, including 13 in which only the participant was interviewed, 23 in which the participant and a surrogate were interviewed, and 34 in which only a surrogate was interviewed. Nine surrogates for patients who died completed the survey. In total, there was a 25.9% response rate amongst living patients with accurate contact information and a 43.5% response rate amongst surrogates with available contact information (Fig. 1).

Descriptive characteristics of those surveyed demonstrates that a majority of respondents were white, and the median ages for both subgroups were similar. (Table 1) There were significantly more male patients versus surrogates (61.1% vs 21.1%, *p* < 0.001). Of those surrogates surveyed, 38.6% were mothers of the patient (*n* = 22), 7% father (*n* = 4), 26.3% son/daughter (*n* = 15), and 19.3% spouse (*n* = 11). There were no parents of minors surveyed as the PAMPer trial only enrolled patients over 17 years of age. Over 90% of subjects graduated high school, and more than half had annual family incomes over $40,000. About half of the respondents worked full-time, and 12.9% were disabled. More surrogates versus patients were employed full or part time (*p* < 0.001) and surrogates had significantly higher annual family incomes versus patients (*p* = 0.031). The majority of subjects have medical insurance coverage, and a few had previously been involved in medical research. The median time between this telephone interview and enrollment in the PAMPer trial was 3.5 years. Enrolled patients overall had high injury severity scores based upon inclusion criteria of the trial.

**Table 1:** Survey Participants Demographics and Injury Severity

	All Respondents (*N* = 93)	Patient Group (*N* = 36)	Surrogate Group (*N* = 57)	*P*-value
**Male sex—no. (%)**	34 (36.6)	22 (61.1)	12 (21.1)	**< 0.001**
**Race—no. (%)**				
**Not reported**	3 (3.2)	1 (2.8)	2 (3.5)	0.827
**Asian**	2 (2.2)	1 (2.8)	1 (1.8)	
**Black**	1 (1.1)	0 (0.0)	1 (1.8)	
**Hispanic**	2 (2.2)	0 (0.0)	2 (3.5)	
**Multicultural**	3 (3.2)	1 (2.8)	2 (3.5)	
**White**	82 (88.2)	33 (91.7)	49 (86.0)	
**Median age (IQR)—years**	52 (39–61)	50 (33–57)	54 (43–61)	0.101
**Median hospital length of stay (IQR)—days**	20 (8–30)	21 (7.8–31)	19.5 (8–29.3)	0.830
**Median length of mechanical ventilation (IQR)—days**	5 (1–13.8)	4.5 (0–12.3)	6.5 (1.8–15)	0.324
**Median Injury Severity Score (IQR)**	22 (13–33)	20.5 (12–33.3)	22 (13–30.8)	0.916
**Injury caused by blunt trauma—no. (%)**	77 (91.7)	31 (91.2)	46 (92.0)	0.893
**Prehospital intubation—no. (%)**	25 (29.8)	5 (14.7)	20 (40.0)	**0.013**
**Procedures in 1st 24 h—no. (%)**	59 (70.2)	24 (70.6)	35 (70.0)	0.954
**Transported from scene of injury—no. (%)**	64 (76.2)	25 (73.5)	39 (78.0)	0.637
**Median time between interview and enrollment (IQR)—years**	3.5 (2.3–4.0)	3.24 (2.2–3.9)	3.5 (2.3–4.1)	0.636
**Education level —no. (%)**				
**Not reported**	3 (3.2)	1 (2.8)	2 (3.5)	0.479
**Less than high school**	1 (1.1)	1 (2.8)	0 (0.0)	
**Some high school**	2 (2.2)	1 (2.8)	1 (1.8)	
**High school graduate**	27 (29.0)	8 (22.2)	19 (33.3)	
**Technical college**	1 (1.1)	0 (0.0)	1 (1.8)	
**Some college**	25 (26.9)	11 (30.6)	14 (24.6)	
**College graduate**	22 (23.7)	7 (19.4)	15 (26.3)	
**Some postgraduate**	1 (1.1)	0 (0.0)	1 (1.8)	
**Postgraduate**	11 (11.8)	7 (19.4)	4 (7.0)	
**Employment status—no. (%)**				
**Not reported**	3 (3.2)	1 (2.8)	2 (3.5)	**< 0.001**
**Disabled**	12 (12.9)	10 (27.8)	2 (3.5)	
**Employed full-time**	46 (49.5)	11 (30.6)	35 (61.4)	
**Employed part-time**	6 (6.5)	1 (2.8)	5 (8.8)	
**Employed part-time, student**	1 (1.1)	1 (2.8)	0 (0.0)	
**Not employed**	4 (4.3)	4 (11.1)	0 (0.0)	
**Retired**	18 (19.4)	5 (13.9)	13 (22.8)	
**Seasonal**	1 (1.1)	1 (2.8)	0 (0.0)	
**Student**	2 (2.2)	2 (5.6)	0 (0.0)	
**Annual family income (before taxes) —no. (%)**				
**Not reported**	6 (6.5)	4 (11.1)	2 (3.5)	**0.031**
**<$5000**	7 (7.5)	2 (5.6)	5 (8.8)	
**$5000–$19,999**	6 (6.5)	6 (16.7)	0 (0.0)	
**$20,000–$39,999**	23 (24.7)	9 (25.0)	14 (24.6)	
**$40,000–$59,999**	12 (12.9)	3 (8.3)	9 (15.8)	
**$60,000–$79,999**	11 (11.8)	3 (8.3)	8 (14.0)	
**>$80,000**	28 (30.1)	9 (25.0)	19 (33.3)	
**Medical insurance coverage—no. (%)**	89 (95.7)	35 (97.2)	54 (94.7)	0.711
**Previous medical research involvement—no. (%)**	14 (15.1)	8 (22.2)	6 (10.5)	0.124
**Previous refusal of medical research involvement—no. (%)**	6 (6.5)	1 (2.8)	5 (8.8)	0.252

### Effectiveness of community consultation and views of EFIC

**Fig. 2:**
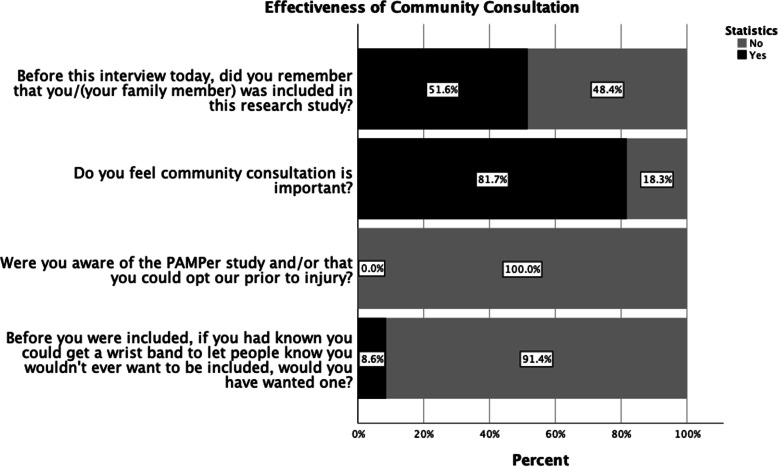
Effectiveness of Community Consultation

Nearly half (48.4%, *n* = 45/93) of respondents did not recall their personal or family member enrollment in PAMPer. The majority of subjects (81.7%) believed public disclosure/community consultation was important prior to EFIC trials. None of the patients or surrogates were informed of the PAMPer trial through public disclosure, and none were aware of opt out procedures. If they hypothetically had known about their ability to opt out via wrist bands, 8.6% would have chosen to opt out (Fig. 2).

**Fig. 3:**
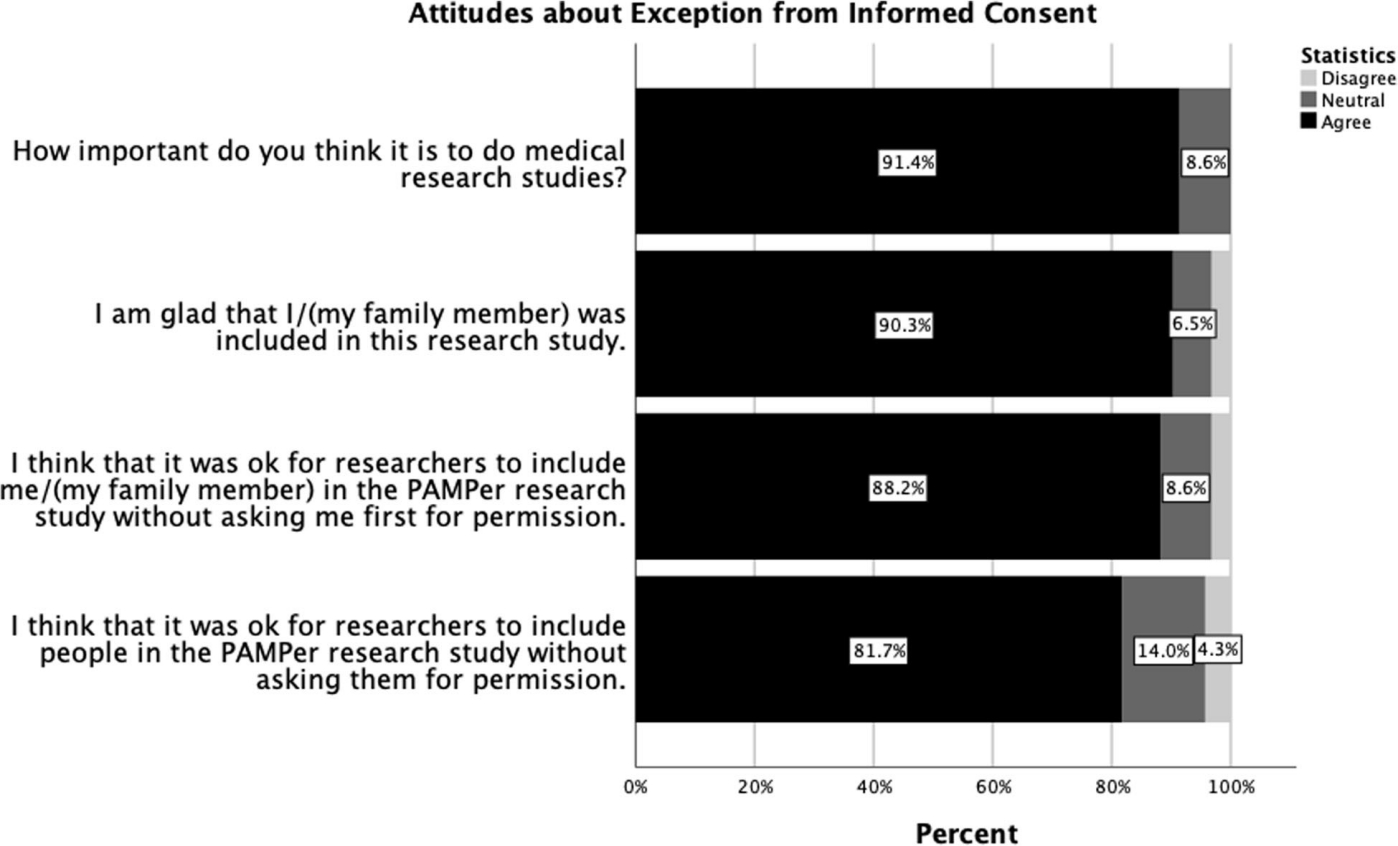
Attitudes about Exception from Informed Consent

Without disclosure of study outcome, patients and surrogates were glad they were enrolled (90.3%), agreed with EFIC use for their personal enrollment (88.2%), and agreed with the general use of EFIC for the PAMPer study (81.7%) (Fig. 3).

Responses from patients and surrogates were not statistically different when asked about the importance of medical research, opinions regarding personal enrollment, EFIC for personal enrollment, and EFIC for general enrollment. Furthermore, responses from surrogates for patients who were alive at the time of the interview (*n* = 48) versus surrogates for patients who died (*n* = 9) were not statistically different for all of these questions.

These results were independent of age, gender, and respondent type.

### Opinions based on study outcome

To assess the way in which the outcome of the trial influenced patient and surrogate opinions related to EFIC, participants were asked to respond to select questions previously asked once again following disclosure of the positive results of the PAMPer study as well as after a hypothetical neutral or negative outcome was proposed.

Disclosure of the survival benefit associated with the PAMPer study resulted in a significant increase in opinions regarding personal enrollment (90.3% vs 94.6%, *p* < 0.001), EFIC for personal enrollment (88.2% vs 94.6% *p* < 0.001), and EFIC for general enrollment (81.7% vs 84.9%, *p* < 0.001). Disclosure of a hypothetical neutral or negative study outcome resulted in significant decreases in opinions regarding EFIC for personal enrollment (88.2% vs 79.6%, *p* = 0.003) and EFIC for general enrollment (81.7% vs 72.0%, *p* < 0.001). Although not significant, there was also a decrease in agreement with EFIC use for personal enrollment with disclosure of a neutral or negative study outcome (90.3% vs 79.6%, *p* = 0.060).

When participants were asked if they agreed with the use of randomization to assign treatment groups, 43.0% of subjects agreed given a positive trial outcome, while 38.7% agreed given a neutral or negative trial outcome (*p* < 0.001).

### Attitudes toward doctors and emergency research

**Fig. 4:**
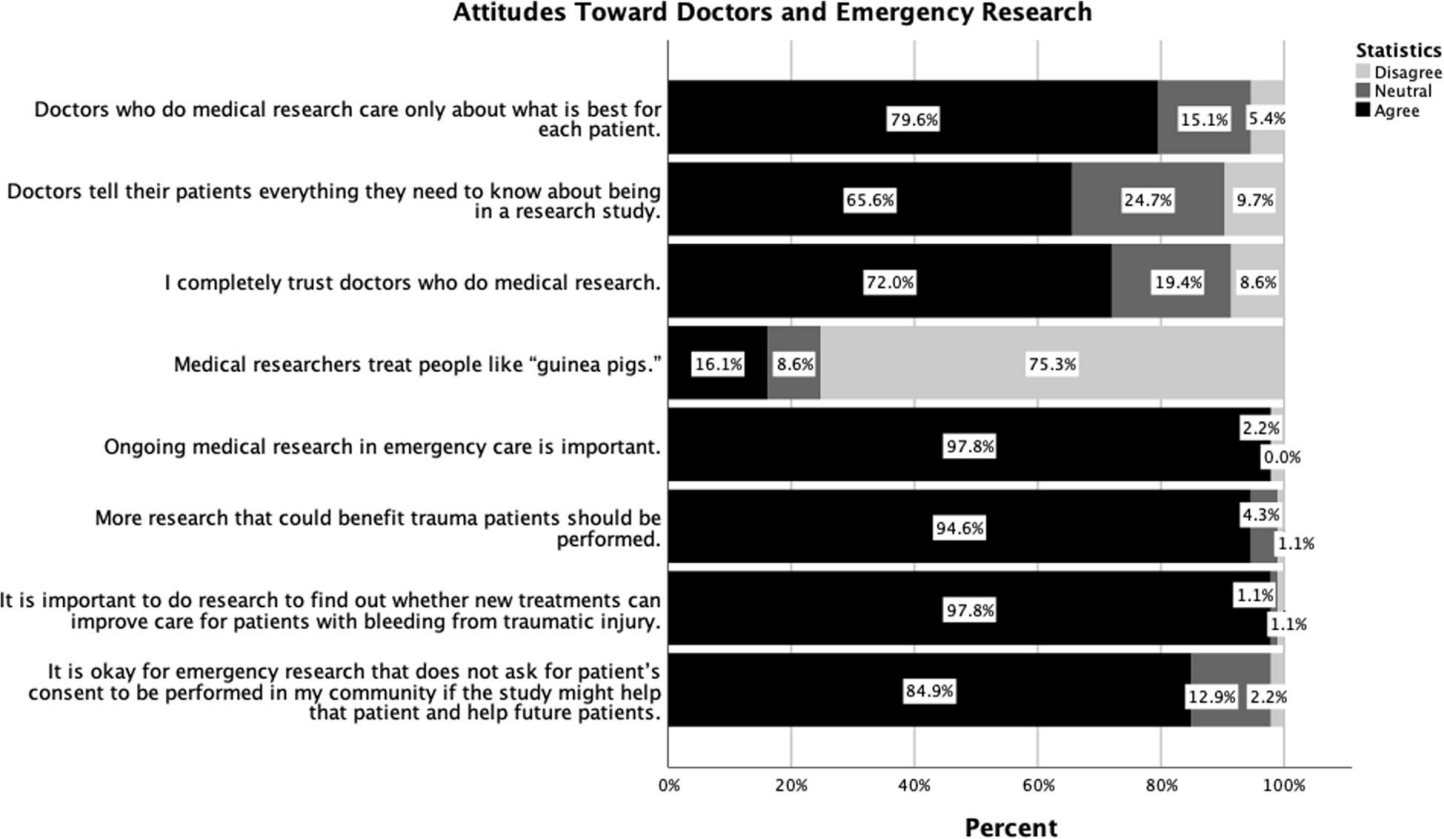
Attitudes Toward Doctors and Emergency Research

Subjects’ attitudes toward medical research and doctors was measured via a series of Likert Scale questions. Relative to all respondents (*n* = 93), 91.4% of subjects believed it was important to do medical research, 79.6% agreed that doctors who do medical research care only about what is best for each patient, and 65.6% agreed that doctors tell their patients everything they need to know about being in a research study. Of those surveyed, 72.0% of subjects reported that they completely trusted doctors who do medical research, and only 16.1% of respondents agreed that medical researchers treat people like “guinea pigs”. When asked if ongoing medical research in emergency care was important, 97.8% agreed, and 94.6% of subjects believed that more research that could benefit trauma patients should be performed. Most subjects (97.8%) believed that it was important to do research to find out whether new treatments can improve care for patients with bleeding from traumatic injury, and 84.9% of respondents agreed that it was ok for emergency research that does not ask for patient’s consent to be performed in their community if the study might help that patient and help future patients (Fig. 4). Patient versus surrogate responses were not statistically different for all questions.

## Discussion

As the emphasis on prehospital and early resuscitation intervention following injury grows, the use of EFIC for research trials will increase. As such, we ought to better understand the patients’ and surrogates’ willingness to be enrolled without consent, evaluate whether attempts to inform the public prior to a trial are likely to result in participants being aware of the trial prior to enrollment, and assess patients’ and surrogates’ recall of being notified of their enrollment in the trial without consent or being asked for consent to continue in the trial.

Public disclosure and community consultation are necessary steps in any trial involving EFIC protocols. Dickert et al. reported that increased community consultation methods were associated with increased acceptance of EFIC and greater recall of study information but lower recall of risks [21]. Others have noted that public canvassing, in-person community consultation, and targeted community outreach to individuals most at risk of inclusion in the trial were the most successful methods and yielded greater acceptance rates [9, 22, 23].. Public disclosure and community consultation for the PAMPer trial was conducted from 2012 to 2013 and included use of radio, newspaper, and random digit dialing to zip codes eligible for enrollment, online websites, YouTube videos, and flyering. We found that while most (81.7%) participants believed community consultation was important, none of the patients or surrogates could recall being informed of PAMPer via community consultation, and none were aware of opt out procedures. Social media has also been reported to be a cost-effective and efficient means of notifying the public [15, 24]. Accordingly, social media has become one of the primary avenues for community consultation for EFIC since the PAMPer study [31].

Previous studies have analyzed the general population’s opinions toward EFIC studies [5–20]. Populations surveyed were generally accepting of EFIC, with higher acceptance of personal enrollment versus general enrollment. Lower acceptability of EFIC was associated with lower education level and older respondent age. Interestingly, those parents interviewed whose children were in a situation at higher risk to involve EFIC (ex. critical care unit patient) were more supportive of this enrollment practice [8]. There have been conflicting reports on how the proximity to violence, socioeconomic status, and injury mechanism have influenced willingness to participate in EFIC research [14, 19]. We found that when patients and surrogates were retrospectively asked their opinions, they were glad they were enrolled (90.3%), agreed with EFIC use for their personal enrollment (88.2%,), and agreed with the general use of EFIC for the PAMPer study (81.7%). This again demonstrates participants’ hesitancy in approving general EFIC enrollment to refrain from speaking on behalf of others but shows that participants are personally more willing to be enrolled via EFIC.

Very few studies have examined patients’ and surrogates’ opinions regarding EFIC procedures and emergency research after they had participated in the clinical trial [23–28]. This is the first of these studies involving trial participants with uncontrolled traumatic hemorrhage. The greater morbidity and mortality rate within this population in comparison to others that have been assessed may affect participants’ assessments of risks and benefits of the study as well as EFIC acceptability. Of note, these previous studies have shown that patients often had a hard time understanding details relevant to the study in which they were enrolled and exhibited poor recall of risks of the study [25, 27]. Kamarainen et al. found that the patient outcomes did not affect the willingness of consent providers to respond to the survey or how they felt about emergency research [28]. However, Whitesides et al. found that agreement with personal enrollment in the EFIC study was significantly higher among participants with favorable outcomes compared to those with unfavorable outcomes. They also reported that there was a statistically significant relationship between more severe initial injury and increased acceptance of personal or general EFIC enrollment in the study [30]. Validating Kamarainen et al.’s findings, our comparisons of responses from surrogates for patients who were alive at the time of the interview versus surrogates for patients who died demonstrate that patient outcomes did not affect surrogate opinions; however, the small sample size of surrogates interviewed for patients who died (*n* = 9) may impact the validity of these results.

While some studies have looked at the difference in opinions based on patient outcomes, none have examined views regarding EFIC enrollment based on the outcome of the trial in which they participated. While EFIC regulations require that the outcome of the study be disclosed to the public, many of the patients enrolled are not informed about the results of the study in which they participated. We found that disclosure of the 9.8% mortality benefit associated with the PAMPer study resulted in a significant increase in opinions regarding EFIC enrollment, and disclosure of a hypothetical neutral or negative study outcome resulted in significant decreases in opinions. We found a significant decrease in participant agreement with the use of randomization to assign control and treatment groups given a positive versus neutral/negative trial outcome. This verifies that study outcome affects patient or surrogate opinions.

### Limitations

There are limitations to the current study. Response rates pose a problem as this study requires subjects to both answer the phone and agree to participate in the study. Previous studies have had response rates that range from 1.5 to 92%. To minimize this upfront, our cohort was expanded to include both patients and surrogates. Our 25.9% patient response rate and 43.5% surrogate response rate were among the highest previously reported. Due to patient mortality and the gravity of the traumatic events that occurred, we had a large population of patients and surrogates who chose not to participate—contributing to a significant response bias. We had a total of 93 interviews; this relatively small sample size can affect the validity of our results. Our cohort consisted of a large proportion of females, white, and blunt injury; because these demographics could affect willingness to participate in this survey and approval of EFIC enrollment, our results may not accurately reflect opinions of the general population. Given the extended time since patient enrollment in the study, there was significant recall bias, which could affect subjects’ recall of public disclosure/community consultation efforts as well as opinions related to EFIC enrollment. Our survey primarily utilized close-ended questions that focused on an important, yet narrow, slice of the patients’ and surrogates’ overall experience with EFIC. Although survey questions were derived from previously published questions from other surveys, cognitive pre-testing of our survey was not performed, potentially predisposing to misunderstood wording and unwarranted suppositions. While the PAMPer trial brought a known in-hospital resuscitation fluid to the prehospital setting, other EFIC trials that more significantly differ from standard care may have different degrees of acceptability.

## Conclusions

Although EFIC trials are becoming more common, the community may be less informed about the process. We ought to continue to evaluate the efficacy of public disclosure and community consultation and perhaps require this as part of EFIC trial protocol to ensure continued improvements. Despite this, patients and surrogates of patients previously enrolled via EFIC were generally accepting of this type of enrollment procedure. Patients’ and surrogates’ responses were similar, suggesting that surrogates may be excellent proxies for these types of inquiries. Patient outcomes did not affect surrogate opinions. However, the outcomes for the trial in which EFIC was utilized significantly affected patient and surrogate agreement with personal and general EFIC enrollment, with positive outcomes increasing acceptability and negative study outcomes decreasing acceptability. These findings reinforce and extend prior observations from those enrolled with EFIC and show that these can be generalized to this new and important patient population.

## Supplementary information

**Additional file 1.** Survey.

## Data Availability

All data generated or analyzed during this study are included in this published article and its supplementary information files.

## Abbreviations

PAMPer: Prehospital Air Medical Plasma Trial
EFIC: Exception from informed consent
US: United States
WA: Washington
NY: New York
