# Supplementary material for: Patient and surrogate attitudes via an interviewer-administered survey on exception from informed consent enrollment in the Prehospital Air Medical Plasma (PAMPer) trial
Source: BMC Emerg Med. 2020 Oct 1;20:76. doi: 10.1186/s12873-020-00371-6 (PMC7528275; doi:10.1186/s12873-020-00371-6)
Supplement: Supplementary file 1 — Additional file 1. Survey. [file 12873_2020_371_MOESM1_ESM.docx]

1. Before we begin with some questions about your opinion about participation in the study, may I ask your age? **[INFO: IF UNDER 18 ASK TO SPEAK TO SOMEONE OVER 18] [IF AGE <18 THANK AND POLITELY DISCONTINUE]**

The following questions are only to learn more about the demographic of patients we are surveying. Your answers will be kept anonymous.

2. What is your race?

1. White, non-Hispanic

2. Black, non-Hispanic

3. Alaskan Native or American Indian, non-Hispanic

4. Asian or Pacific Islander, non-Hispanic

5. Spanish or Hispanic, any race

6. Multicultural (parents represent different racial ethnic groups)

7. Other **[SPECIFY]**

8. Refused

9.Don't know

3. What is the zip code where you live?

4. What is the last grade or year that you completed in school?

1. Less than high school
2. Some high school
3. High school graduate or GED
4. Some college
5. College graduate
6. Some post-graduate
7. Post-graduate or Professional degree
8. Other, specify __________________________________

5. Which of the following best describes your employment or student status?

Are you employed? (Read options out loud.)

1. Employed Full Time
2. Employed Part Time
3. Retired
4. Not Employed
5. Disabled
6. Student

6. Which category best describes your combined family income in the last year (before taxes)? Was it

1. < $5,000
2. $5,000 - $19,999
3. $20,000 - $39,999
4. $40,000 - $59,999
5. $60,000 - $79,999
6. > $80,000
7. Don’t know

7. Do you currently have medical insurance coverage of any sort?

1. Yes
2. No
3. Don't know
4. Refused

(2) Now I am going to ask you a few questions about your prior research experience and general attitude toward research.

8. Have you ever been a part of a medical research study other than the study we are talking about today? **PROBE:** This could be a study of medications or procedures or could involve answering questions like you're doing today.

1. Yes
2. No **IF NO, SKIP to 10**

9. If so, what kind(s) of study(ies) and what was involved for you?

10. Have you ever been asked to be a part of a research study and decided not to be in the study?

1. Yes
2. No **IF NO, SKIP to 13**

11. What kind(s) of study(ies) and what would have been involved for you?

12. What was your reason for choosing not to be a part of that study?

13. On a scale of 1 to 5 with 1 being very important and 5 being not important at all, how important do you think it is to do medical research studies?

14. We're interviewing you today because you/(your family member) were/was included in a research study for people injured and at risk of bleeding. Before this interview today, did you remember that you/(your family member) were/was included in this research study?

1. Yes
2. No **IF “NO” or “uncertain,” SKIP to 16.**

15. How did you find out that you/(your family member) were/was included in this research study?

16. On a scale of 1 to 5 with 1 being strongly agree and 5 being strongly disagree, how does the following statement apply to you: I am glad that I/(your family member) was included in this research study.

As I mentioned, the study you/(your family member) were/was included in is called the PAMPer study, which compared people with bleeding after a traumatic injury who received plasma in the helicopter to people who did not. Normally, people are only included in research studies after they (or someone who makes decisions for them) have been asked for permission to be included in the study. But because you/(your family member) were/was experiencing heavy blood loss and potentially shock, you didn't find out about your/(your family member) being included in the PAMPer study until afterwards.

17. On a scale of 1 to 5 with 1 being strongly agree and 5 being strongly disagree, how would you rate the following statement? I think that it was ok for researchers to include you/(your family member) in the PAMPer research study without asking me first for permission.

18. Not thinking specifically about your/(your family member)'s situation, I now want to ask you about your thoughts, in general, on whether it was ok for researchers to do this study where people are included without being asked for permission first. On a scale of 1 to 5 with 1 being strongly agree and 5 being strongly disagree, how would you rate the following statement? I think that it was ok for researchers to include people in the PAMPer research study without asking them for permission.

19. As I described earlier, when researchers want to do a study like this where people have to be included without being asked, the rules of research require researchers to ask members of the community before starting the study for their thoughts about the study. In other words, the researchers have to ask the community for input on the study the researchers want to do. Does this seem like something important to do?

1. Yes
2. No

20. These researchers also tried to inform people in the community about the PAMPer study so that they would know it was going on. Were you aware of the PAMPer study, the one in which you/(your family member) were/was enrolled, before you/(your family member ) were/was brought to the hospital?

1. Yes
2. No **(IF NO, SKIP to 22)**

21. (If yes), how did you hear about the study?

22. Were you aware that you could call in and get a wrist band that would let people know you didn't want to be included in this study if you had traumatic injury and were bleeding and were picked up by the helicopter/ambulance?

1. Yes
2. No(**IF NO, SKIP TO 24)**

23. (If yes), did you call? Why or why not?

24. **DON’T ASK IF YES TO QUESTION 22** Before you were included, if you had known you could get a wrist band to let people know you wouldn't ever want to be included, would you have wanted one?

1. Yes
2. No

I want to ask you some questions now about your thoughts about doctors and researchers. On a scale of 1 to 5 with 1 meaning you strongly agree and 5 meaning you strongly disagree, how much would you say you agree with the following statements?

25. Doctors who do medical research care only about what is best for each patient.

26. Doctors tell their patients everything they need to know about being in a research study.

27. I completely trust doctors who do medical research.

28. Medical researchers treat people like “guinea pigs.”

29. Ongoing medical research in emergency care is important.

30. More research that could benefit trauma patients should be performed.

31. It is important to do research to find out whether new treatments can improve care for patients with bleeding from traumatic injury.

32. It is okay for emergency research that does not ask for patient’s consent to be performed in my community if the study might help that patient and help future patients.

The PAMPer study has now been completed and the results were recently published. We found that the group that received plasma had a 10% lesser chance of death when compared to the group that did not receive plasma in the helicopter.

Given this, I have a few more questions related to your opinion on participation in the study. Once again, on a scale of 1 through 5, 1 being strongly agree and 5 being strongly disagree, how much would you say you agree with the following statements?

1. I am glad that I/my family member was included in this research study.
2. I think it was ok for researchers to include me/my family member in the PAMPer research study without asking for permission first.
3. I think that it was ok for researchers to include people in the PAMPer research study without asking them for permission first.
4. I think that it was acceptable for researchers to give half of the patients in the PAMPer study plasma in the helicopter and not give it to the other half in the helicopter.

If this study had instead shown that those patients who received plasma were no better off than those who did not OR if they actually had worse outcomes, how would your opinion change, if at all, to the following questions? Please answer on a scale of 1 through 5, 1 is strongly agree and 5 is strongly disagree for the following questions.

1. I am glad that I/my family member was included in this research study.
2. I think it was ok for researchers to include me/my family member in the PAMPer research study without asking for permission first.
3. I think that it was ok for researchers to include people in the PAMPer research study without asking them for permission first.
4. I think that it was acceptable for researchers to give half of the patients in the PAMPer study plasma in the helicopter and not give it to the other half in the helicopter.

That concludes our survey. You have been very helpful. If you have additional questions or concerns about either this study or the PAMPer study, you can call the University of Pittsburgh Human Subject Protection Advocate at 1-866-212-2668. Thank you very much for your time and cooperation.
